# Toward molecular imaging of the free fatty acid receptor 1

**DOI:** 10.1007/s00592-017-0989-7

**Published:** 2017-04-13

**Authors:** Ewa Hellström-Lindahl, Ola Åberg, Cecilia Ericsson, Gavin O’Mahony, Peter Johnström, Stanko Skrtic, Olof Eriksson

**Affiliations:** 1 Division of Molecular Imaging, Department of Medicinal Chemistry, Uppsala University, SE-751 83 Uppsala, Sweden; 2 AstraZeneca R&D, SE-431 50 Mölndal, Sweden; 3 Personalised Healthcare and Biomarkers, AstraZeneca PET Science Centre, Karolinska Institutet, Karolinska University Hospital, SE-17176 Stockholm, Sweden; 4 Institute of Medicine, Sahlgrenska Academy, University of Gothenburg, SE-413 45 Gothenburg, Sweden

**Keywords:** FFAR1, GPR40, Beta cell imaging, Islet imaging, Drug development

## Abstract

**Aims:**

Molecular imaging of the free fatty acid receptor 1 (FFAR1) would be a valuable tool for drug development by enabling in vivo target engagement studies in human. It has also been suggested as a putative target for beta cell imaging, but the inherent lipophilicity of most FFAR1 binders produces high off-target binding, which has hampered progress in this area. The aim of this study was to generate a suitable lead compound for further PET labeling.

**Methods:**

In order to identify a lead compound for future PET labeling for quantitative imaging of FFAR1 in human, we evaluated tritiated small molecule FFAR1 binding probes ([^3^H]AZ1, [^3^H]AZ2 and [^3^H]TAK-875) for their off-target binding, receptor density and affinity in human pancreatic tissue (islets and exocrine) and rodent insulinoma.

**Results:**

[^3^H]AZ1 showed improved specificity to FFAR1, with decreased off-target binding compared to [^3^H]AZ2 and [^3^H]TAK-875, while retaining high affinity in the nanomolar range. FFAR1 density in human islets was approximately 50% higher than in exocrine tissue.

**Conclusions:**

AZ1 is a suitable lead compound for PET labeling for molecular imaging of FFAR1 in humans, due to high affinity and reduced off-target binding.

## Introduction

Free fatty acid receptor 1 (FFAR1), also known as GPR40, is emerging as an important therapeutic target. It is a G-coupled transmembrane protein, which acts as a nutrient sensor by interacting with medium to long chain fatty acids, in particular eicosatrienoic acid (20:3) and docosahexaenoic acid (22:6) in the blood stream. It has been intricately linked with energy homeostasis, as receptor activation contributes to downstream increase in insulin secretion in the pancreatic beta cells. Synthetic agonists of the FFAR1 are therefore developed as potential therapeutic agents in metabolic disease [1, 2]. FFAR1 is also highly expressed in the brain where it has been linked to neuronal function and pain as well as in taste bud cells acting as a dietary fat sensor.

Molecular imaging of the FFAR1, by for example radioactive ligands for Positron Emission Tomography (PET), would be a valuable tool for drug development by enabling in vivo target engagement studies in human. Additionally, it could contribute to directly assess its regulation during different metabolic states in human.

Natural occurring and synthetically generated ligands for FFAR1 are generally highly lipophilic, since FFAR1 agonism or antagonism involves binding to an inner hydrophobic pocket [3]. Development of direct in vitro assays for FFAR1 binding has therefore been difficult, since lipophilic radiolabeled agents usually exhibit high non-specific (off-target) interactions, which may mask the receptor bound signal. Indirect readouts, such as functional activity in cells, have usually been used instead. Only recently have assays utilizing fluorescently labeled reporter probes with decreased off-target binding been reported [4–6]. Only one FFAR1 targeting PET ligand (potentially making possible in vivo imaging in human, the relevant setting) has been reported [7].

In this study, we examined radiolabeled small molecule FFAR1 binding probes for their off-target binding in human pancreatic tissue, in order to identify a lead compound for future PET labeling for quantitative imaging of FFAR1 in human.

## Method and materials

### Chemicals

**Fig. 1:**
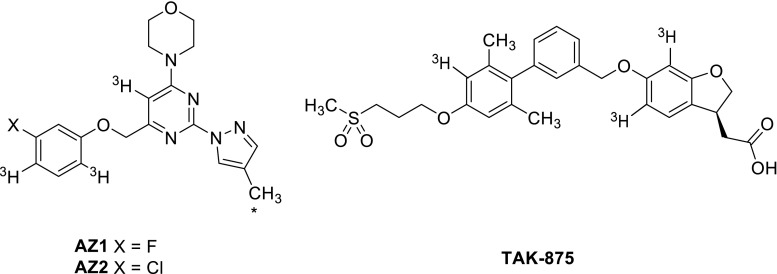
Structures and labeling positions of AZ1, AZ2 and TAK-875

FFAR1 agonists [^3^H]AZ13263340 ([^3^H]AZ1, specific activity 18.83 Ci/mmol) (Fig. 1), [^3^H]AZ13253035 ([^3^H]AZ2, specific activity 25.38 Ci/mmol) (Fig. 1), [^3^H]TAK-875 (specific activity 66.7 Ci/mmol) (Fig. 1) and their unlabeled analogs were synthesized by AstraZeneca R&D, Mölndal, Sweden.

### Proof of binding of AZ1, AZ2 and TAK-875 to FFAR1

The compounds have been evaluated in a functional assay using mouse GPR40 receptor overexpressing HEK293 cells and the IP-One Tb HTRF technology (data on file, AstraZeneca). The obtained EC_50_ values were: 36.5 nM (AZ1); 12.1 nM (AZ2); >37.5 μM (TAK-875).

### Tissues for binding studies

Rat insulinoma cell line INS-1 xenografts, explanted from immunodeficient mice postmortem, were used as a model of beta cells. Briefly, approximately 2 million INS-1 cells were injected subcutaneously in the right flank of Balbc nu/nu mice. Tumor growth was monitored by palpation, and when tumor diameter was 10 mm, the animals were euthanized and the INS-1 tumor explanted.

Isolated pancreatic islets and exocrine tissue were obtained within the Nordic network for Clinical Islet Transplantation laboratory in Uppsala, Sweden. The use of human tissue was approved by the Uppsala Ethical Review Board (#2011/473, #Ups 02-577).

### Preparation of tissue for in vitro binding studies

Isolated endocrine (75–95% islet purity) and exocrine tissues were homogenized in ice-cold 0.32 M sucrose by hand using a Dounce glass homogenizer to a final concentration of 6 mg/ml. 50–100 mg INS-1 xenografts were homogenized using a Polytron tissue homogenizer (Polytron^®^ PT 3000, Kinematica AG, Littau-Luzern, Switzerland) in ice-cold 0.32 M sucrose at a concentration of 6 mg/ml and then by hand using a Dounce glass homogenizer. Protein concentration was determined using Bio-Rad Protein Assay (Bio-Rad Laboratories, Hercules, CA) with bovine serum albumin as standard. Aliquots of the homogenates were stored at −80 °C until used.

### In vitro binding assay for [^3^H]AZ1, [^3^H]AZ2 and [^3^H]TAK-875

One milligram of homogenized endocrine and exocrine tissue and 2 mg insulinoma tissue was incubated for 2 h at room temperature with 2 nM [^3^H]AZ1 or 2 nM [^3^H]AZ2 and PBS in a final incubation volume of 1 ml. Two milligram insulinoma tissue was incubated for 2 h at room temperature with 4 nM [^3^H]TAK-875 and 50 mM TRIS (pH 7.4) in a final incubation volume of 1 ml. All three ligands were dissolved in ethanol. 10 µM of unlabeled AZ1 or TAK-875 was added for determination of non-specific binding. The samples were filtered using a Brandel M-48 cell harvester with Whatman GF/C filter (presoaked with PBS or 50 mM TRIS) and washed four times with 3 ml PBS or 50 mM TRIS (room temperature). Filters were put into scintillation vials, dried for 30 min before adding 10 ml Ultima Gold scintillation fluid (Perkin Elmer, Waltham, MA). The filters were shaken for 60 min and then counted in a *β*-counter (Packard Scintillator Tri-Carb 2100TR). The *K*_d_ and *B*_max_ values for [^3^H]AZ1 binding were determined using concentrations of the tritiated ligand from 1 to 20 nM. All assay concentrations were replicated in triplicate, both total binding and non-specific binding.

The specific binding for each concentration of radiolabeled ligand was calculated by subtracting the non-specific binding (average of three replicates) from the total binding (average of three replicates), and expressed as fmol or pmol/mg protein. The specific binding data were analyzed with curve-fitting software (GraphPad Prism version 5.04) to obtain *K*_d_ and *B*_max_ values.

### Statistical analysis

When reported on group level, results are given as mean ± SEM. Differences between groups were assessed by one-way ANOVA with Tukey’s post hoc multiple comparisons test using a 95% confidence level.

## Results

### Off-target interactions decrease with decreasing lipophilicity

**Fig. 2:**
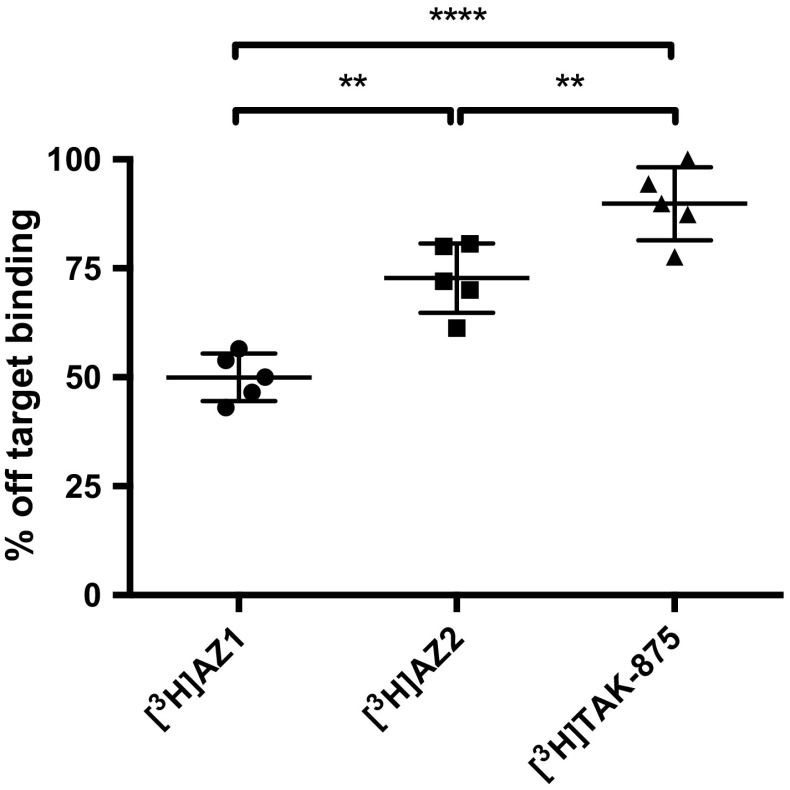
Decreased off-target (non-displaceable) binding of [^3^H]AZ1 (2 nM, clogP = 3.6) and [^3^H]AZ2 (2 nM, clogP = 4.1) compared to [^3^H]TAK-875 (4 nM, clogP = 4.2), in INS-1 xenograft tissue. The three agents exhibited different degrees of off-target binding, with [^3^H]AZ1 having least and [^3^H]TAK-875 having most off-target binding, correlating to their decreasing lipophilicity

[^3^H]TAK-875 (fasiglifam), which is a small molecule FFAR1 agonist that reached clinical phase 3, exhibited high non-specific off-target binding in insulinoma xenograft tissue, demonstrating the difficulties involved in molecular imaging with lipophilic compounds (Fig. 2). Off-target binding was decreased for both [^3^H]AZ1 and [^3^H]AZ2, paralleling their decrease in lipophilicity (*R* ^2^ = 0.79, *p* < 0.0001). [^3^H]AZ1 exhibited approximately 50% specific binding at the incubation dose given here (2 nM).

### Improved measurement of FFAR1 with [^3^H]AZ1 and [^3^H]AZ2

**Fig. 3:**
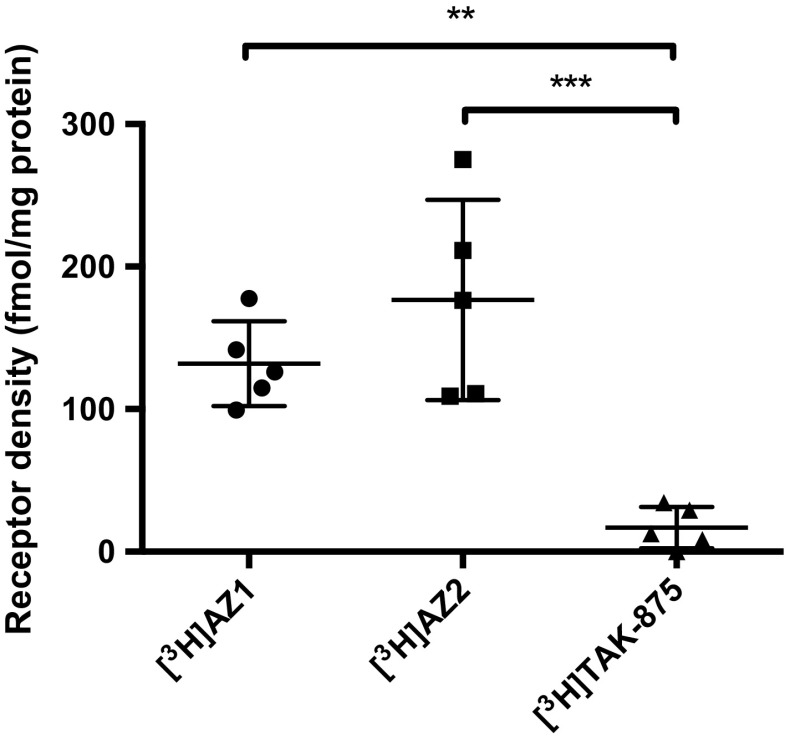
Assessment of FFAR1 density in INS-1 xenograft tissue. [^3^H]TAK-875 could not be reliably used for assessment of FFAR1 receptor density due to its high off-target binding masking the FFAR1 mediated signal. [^3^H]AZ1 and [^3^H]AZ2 on the other hand yielded receptor densities in a similar range

Measurement of FFAR1 density in INS-1 xenograft tissue (by quantifying fmol specific bound radiolabeled ligand per mg of tissue normalized to protein content) was severely underestimated by [^3^H]TAK-875, due to the high masking off-target binding (Fig. 3). [^3^H]AZ1 and [^3^H]AZ2 bound to FFAR1 by 132 and 177 fmol/mg protein, respectively, in this rodent derived beta cell model, at incubation doses of 2 nM radiolabeled ligand.

### FFAR1 density in human islets of Langerhans and exocrine tissue

**Fig. 4:**
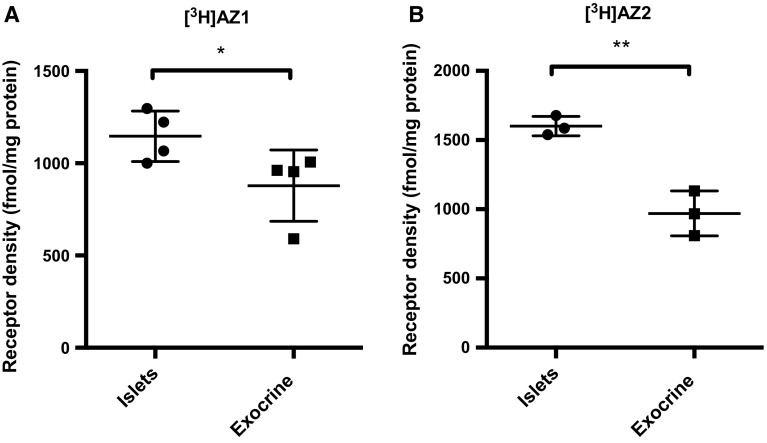
**a** Assessment of FFAR1 density in different human pancreatic compartments. **b** [^3^H]AZ1 at 2 nM showed 30% higher FFAR1 binding in human islets compared to human exocrine tissue (*p* < 0.05). The readout from [^3^H]AZ2 at 2 nM was similar to that of [^3^H]AZ1, but with a more pronounced binding in human islets (*p* < 0.05). The binding of [^3^H]AZ2 in human islets was approximately 50% higher than in exocrine tissue (*p* < 0.01)

FFAR1 density in isolated human islets of Langerhans was estimated to approximately 1150 fmol/mg protein by [^3^H]AZ1 binding (2 nM labeled ligand) (Fig. 4a). The binding estimation was somewhat higher, approximately 1600 fmol/mg protein for [^3^H]AZ2 (2 nM labeled ligand) (Fig. 4b).

We also found relevant amounts of receptor specific binding in human pancreatic exocrine tissue, just below 1000 fmol/mg protein, for both 2 nM [^3^H]AZ1 and [^3^H]AZ2. Still, there was higher binding in islets of Langerhans compared to exocrine tissue for both [^3^H]AZ1 (*p* < 0.05) as well as for [^3^H]AZ2 (*p* < 0.01), with an islet-to-exocrine ratio of 1.3 and 1.65, respectively, at the nanomolar range.

### *B*_max_ and affinity of lead compound [^3^H]AZ1 in pancreas

Based on the off-target binding profile, [^3^H]AZ1 was deemed as the most promising FFAR1 imaging ligand going forward. Affinity (as the dissociation constant *K*_d_) and *B*_max_ measurements, by saturation binding studies, were therefore only performed on [^3^H]AZ1 due to scarcity of human pancreatic tissue.

**Fig. 5:**
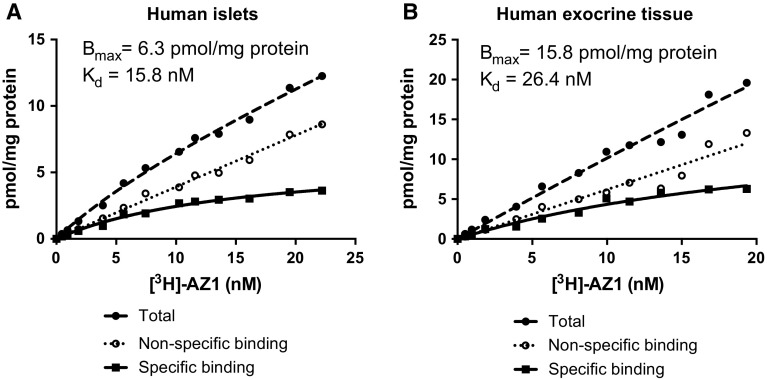
Representative saturation binding experiments of [^3^H]AZ1 performed in **a** human islet preparations and **b** human exocrine preparations. Specific binding for each concentration was calculated by subtracting the non-specific (average of three replicates) from the total binding (average of three replicates)

The affinity of [^3^H]AZ1 in human islets of Langerhans was 10.6 ± 1.5 nM (*n* = 3), which is in the desired range for an in vivo PET ligand (Table 1, representative saturation binding experimental data in Fig. 5a). A similar affinity was found in the exocrine pancreas (19.7 ± 8.4 nM, *n* = 5), likely representing a similar binding site (Table 1, representative saturation binding experimental data in Fig. 5b).

**Table 1:** Assessment of *K*_d_ and *B*_max_ of [^3^H]AZ1 in islet and exocrine tissue homogenates by saturation binding experiments

	Islets (*n* = 3)	Exocrine (*n* = 5)	
*K*_d_ (nM)	10.6 ± 1.5	19.7 ± 8.4	n.s.
*B*_max_ (pmol/mg protein)	7.0 ± 2.0	13.6 ± 2.5	n.s.

The receptor density *B*_max_ was similar in human islets of Langerhans and human exocrine tissue (7.0 vs. 13.6 pmol/mg protein, *p* = 0.13). The higher averaged value in exocrine tissue was mainly due to one outlier with very high uptake.

## Discussion

We here identify [^3^H]AZ1 as a lead compound for further development of a FFAR1 PET imaging agent, based on suitable affinity in the nanomolar range in pancreas and decreased off-target binding.

TAK-875 (fasiglifam) was the first FFAR1 compound to enter clinical phase. It demonstrated promising therapeutic effects before being withdrawn during phase 3 trials based on suspected hepatic toxic side effects at oral dosing of 50 mg daily in individuals with Type 2 diabetes with cardiovascular risk factors (NCT01609582). The delicate balance between retaining drug exposure in the therapeutic window while minimizing unwanted side effects is a common theme in drug development. Quantitative in vivo methodologies for estimating target engagement or receptor occupancy of study drug candidates in target tissues in early clinical phase provides a unique opportunity for assisting in optimizing dosage to achieve the right exposure in the intended target organ. One can thus identify poor candidate drugs before one has to commit to resource intensive late clinical phases and exposing study subject to avoidable drug testing. In the case of FFAR1, the target tissue is the pancreas located deep inside the body. Therefore, the only molecular imaging techniques in question are PET or Single Photon Emission Computed Tomography (SPECT).

In this study, we were interested in developing a lead compound for PET labeling. As expected, the lipophilicity of the ligands was an issue. The high lipophilicity is inherently an issue for FFAR1, since its synthetic ligands are designed to bind with high potency to the same site as long fatty acids. It is likely that there is a trade-off where increasing hydrophilicity (with lower off-target binding) leads to lower affinity to FFAR1 itself.

[^3^H]TAK-875 is highly lipophilic (clogP 4.2, as calculated with MarvinSketch (ChemAxon, Budapest, Hungary)), and consequently the off-target binding of this radiolabeled ligand risk masking the receptor specific bound signal as seen here. [^3^H]AZ1 (clogP 3.6) in particular seemed to retain close to nanomolar FFAR1 affinity while simultaneously reducing the off-target binding substantially compared to [^3^H]AZ2 (clogP 4.1) and [^3^H]TAK-875 (clogP 4.2). Importantly, there was little difference in non-specific binding of [^3^H]AZ1 in different pancreatic compartments, which is a requirement for quantitative molecular imaging.

FFAR1 has previously been reported as being relatively beta cell selective [2]. High protein expression in beta cells, combined with absence in the exocrine pancreas, is one of the hallmarks required for putative beta cell imaging agents [8], and FFAR1 was therefore proposed as a possible surrogate imaging biomarker for the human beta cell. However, expression in alpha cells in mouse has also been reported [9]. However, FFAR1 agonism in human islets does not seem to induce glucagon secretion in a biologically significant amount [10]. Here, we found similar FFAR1 density in another model of pure beta cells (insulinoma xenografts) as in islet homogenates. This indicates that FFAR1 expression also is present in the alpha cells (the other main cell constituent of the islets), since otherwise the islet signal should be decreased (when normalized for volume of tissue).

Unexpectedly, we found significant amount of receptor-mediated binding also in exocrine pancreatic preparations, both using [^3^H]AZ1 and [^3^H]AZ2. The binding was high enough to exclude endocrine contamination of the preparations. Such a high exocrine signal would possibly impede the use of FFAR1 molecular imaging for visualization of the islet of Langerhans in the human pancreas. Here, we measure an islet-to-exocrine binding ration of approximately 1.5, i.e., FFAR1 density is 50% higher in islets of Langerhans. If this translates in vivo it will be highly difficult or impossible to isolate the islet specific signal as it only accounts for up 2% of the total pancreas volume. An islet-to-exocrine ratio upward 50 is required for visualization of the islet mass, and with these results in mind FFAR1 is likely not a good surrogate imaging biomarker.

On the other hand, a relatively poor islet-to-exocrine ratio would not affect the possibility of measurement of receptor occupancy of putative candidate drugs, as the FFAR1-mediated binding of study drugs should be similar in all pancreatic compartments anyway. In vivo target engagement studies of FFAR1 in the pancreas are therefore still a viable approach, regardless of receptors in the exocrine compartment.

The first FFAR1 targeting ligand labeled with Fluorine-18 (a positron emitting nuclide) was recently reported [7]. The radiolabeled probed retained high potency for activation of FFAR1. However, since the probe was a close analog of TAK-875, its lipophilicity remained high (log *p* = 4.58 as calculated by Marwin Sketch, even more lipophilic compared to clogP 4.2 for TAK-875) and it is likely that off-target binding will pose an issue for in vitro and in vivo imaging. Increased uptake in FFAR1 overexpressing HEK cells, compared to native HEK cell, was shown, but no blocking studies (e.g., co-incubation with unlabeled TAK-875 in excess) were reported to separate a FFAR1 receptor specific signal from the presumably high off-target binding.

We have here reported AZ1 as a lead compound toward molecular imaging of FFAR1, due to lower off-target binding compared to other candidates while retaining high affinity and potency, both prerequisites for PET imaging.

Recent research in radiolabeling methodology has expanded the scope of ^11^C-methylations beyond the conventional nucleophilic substitution on heteroatoms such as nitrogen, sulfur and oxygen to include also transition metal mediated ^11^C-methylations on aromatic carbons [11, 12]. In this sense, AZ1 and AZ2 could potentially be ^11^C-labeled at the aromatic methyl position using [^11^C]methyl iodide and the corresponding pyrazol eboronic acid pinacol ester via the palladium mediated Suzuki cross-coupling reaction [13]. A closely related reaction is the Stille coupling using the corresponding aryltributylstannane precursor [14].

Regarding safety considerations, TAK-875 displayed suspected hepatic toxic side effects in a population of 5000 patients, at sustained (years) of oral dosing of 50 mg daily. A clinical PET scan using radiolabeled AZ1 would by definition entail intravenous dosing of microdose levels of AZ1 (<1 µg). We do not foresee any comparable toxicity issues for a single dose of PET ligand in the microdosing interval.

Both [^3^H]AZ1 and [^3^H]AZ2 show improved specificity to FFAR1, with decreased off-target binding compared to [^3^H]TAK-875. This is likely due to the decreased lipophilicity, as there was a linear correlation between off-target binding and clogP for these compounds. Additional improvement in off-target binding is conceivable if the clogP can be further decreased with 1–2 log units by chemical modification.

## Conclusion

We describe two radiolabeled FFAR1 agonists with improved off-target binding compared to TAK-875. [^3^H]AZ1 can furthermore be utilized for measurement of FFAR1 receptor density in human pancreatic tissues in vitro. AZ1 is therefore a suitable lead compound for PET labeling for molecular imaging of FFAR1 in humans.
